# Adipose tissue specific insulin resistance and prognosis of nondiabetic patients with ischemic stroke

**DOI:** 10.1186/s13098-023-01235-2

**Published:** 2023-12-02

**Authors:** Qi Zhou, Hongyi Yan, Aoming Jin, Xia Meng, Jinxi Lin, Hao Li, Yongjun Wang, Yuesong Pan

**Affiliations:** 1 Department of Neurology, Beijing Tiantan Hospital, Capital Medical University, No.119, South 4th Ring West Road, Fengtai District, Beijing, 100070 China; 2 China National Clinical Research Center for Neurological Diseases, Beijing, China

**Keywords:** Adipose tissue, Insulin resistance, Metabolism, Risk, Stroke

## Abstract

**Background:**

Insulin resistance is linked to atherosclerotic cardiovascular diseases and stroke, whereas less is known about adipose tissue specific insulin resistance and outcomes after ischemic stroke. This study aimed to estimate the association between adipose tissue specific insulin resistance and prognosis of nondiabetic patients with ischemic stroke.

**Methods:**

Patients with ischemic stroke without a history of diabetes mellitus in the Third China National Stroke Registry were included. Adipose tissue specific insulin resistance index (Adipo-IR) was calculated by fasting serum insulin and free fatty acids and categorized into 5 groups according to the quintiles. Outcomes included stroke recurrence (ischemic or hemorrhagic), combined vascular events, all-cause death, and poor outcome (modified Rankin Scale, 3–6) at 12 months after stroke onset. We assessed the association between Adipo-IR and risk of prognosis by multivariable Cox/logistic regression models adjusted for potential covariates.

**Results:**

Among 2,222 patients, 69.0% were men with a mean age of 62.5 years. At 12 months, 185 (8.3%) patients had recurrent stroke, 193 (8.7%) had combined vascular events, 58 (2.6%) died, and 250 (11.5%) had a poor outcome. Compared with patients with the lowest quintile, patients with the second, third, fourth, fifth quintiles of the Adipo-IR were associated with an increased risk of stroke recurrence (hazard ratio [HR], 1.77; 95% CI, 1.04–3.03; *P* = 0.04; HR, 2.19; 95% CI, 1.30–3.68; *P* = 0.003; HR, 1.84; 95% CI, 1.06–3.21; *P* = 0.03; HR, 2.11; 95% CI, 1.20–3.71; *P* = 0.01, respectively) and marginally associated with an increased risk of combined vascular events ( HR, 1.60; 95%CI, 0.97–2.64; *P* = 0.07; HR, 1.91; 95% CI, 1.17–3.13; *P* = 0.01; HR, 1.62; 95% CI, 0.96–2.75; *P* = 0.07; HR, 1.80; 95% CI, 1.05–3.09; *P* = 0.03, respectively) at 12 months after adjustment for potential covariates. Adipo-IR was not associated with mortality and poor outcome at 12 months.

**Conclusions:**

These findings suggest that adipose tissue specific insulin resistance is independently associated with recurrent stroke and combined vascular events after acute ischemic stroke in nondiabetic patients.

**Supplementary Information:**

The online version contains supplementary material available at 10.1186/s13098-023-01235-2.

## Introduction

Stroke, the most common serious manifestation of cerebrovascular disease, is also the top contributor to disability-adjusted life years in China [1–4]. Patients with ischemic stroke had a recurrence rate ranging from 12.5 to 17.0% at 12 months [5, 6], and more than half of stroke survivors suffered a recurrent stroke within 9 years of follow-up [6]. Several prognostic factors, particularly abnormal glucolipid metabolism, contribute to the recurrence of stroke [7, 8].

Insulin resistance is defined as decreased responsiveness or sensitivity to insulin metabolic activities, such as glucose disposal in skeletal muscle and adipose tissue and inhibition of gluconeogenesis in liver as well as reduced lipogenesis and lipolysis and circulating presence of free fatty acids [9]. Researchers recognize the critical role of insulin resistance in abnormal glucolipid metabolism that can result in vascular inflammation and atherosclerosis, and subsequent stroke recurrence [10, 11]. However, recent studies indicate that insulin resistance is tissue specific, whereas previous investigations on insulin resistance and stroke outcome focused on whole-body insulin resistance, rather than particular tissues [12, 13]. It is reported that adipose tissue is a highly insulin-responsive organ that affects both glucose and lipid metabolism [14]. Insulin resistance in adipose tissue, assessed by the adipose tissue insulin resistance index (Adipo-IR), induces ectopic lipid accumulation [15]. Subsequently, adipose tissue dysfunction accelerates local inflammation, vascular fibrosis, and deficiency of vasculature [16]. A cross-sectional study in China demonstrated that the insulin resistance status of adipose tissue tends to be related to the emergence of type 2 diabetes and cardiovascular disease [17]. Findings from the GEA study that Adipo-IR may promote the progression of aortic valve calcification also supported a potential negative association of Adipo-IR with cardiovascular disease [18]. However, there is a lack of investigation on stroke outcomes in patients with adipose tissue specific insulin resistance in a large longitudinal study. Therefore, the association between adipose tissue specific insulin resistance and stroke prognosis is still unclear.

In the present study, we aimed to determine whether adipose tissue specific insulin resistance is associated with prognosis of nondiabetic patients after acute ischemic stroke in a large-scale, nationwide stroke registry in China.

## Methods

### Study design and participants

The study population was derived from the Third China National Stroke Registry (CNSR-III), a nationwide registry of ischemic stroke or transient ischemic attack (TIA) in China based on etiology, imaging, and biological indicators. Information about the project’s purpose, structure, and methods has previously been published [19]. Researchers enrolled patients in the CNSR-III at 201 hospitals across 22 provinces and four municipalities in China from August 2015 to March 2018. Acute ischemic stroke was diagnosed according to the World Health Organization criteria and confirmed by brain computed tomography or magnetic resonance imaging [20]. The severity of stroke was evaluated by the National Institutes of Health Stroke Scale [21]. Patients were enrolled by face-to-face interview and followed up by telephone interviews at 12 months after acute ischemic stroke/TIA. The study was reported according to the “Strengthening the Reporting of Observational Studies in Epidemiology” (STROBE) guideline [22].

Diabetes was defined as a self-reported previous definite diagnosis of diabetes or a history of antidiabetic drug use or antidiabetic treatment during hospitalization. According to the purpose of this study, we only included nondiabetic patients with ischemic stroke into our study. Among 201 participating hospitals, 171 sites had experience collecting blood samples, which constituted the source of a prespecified subgroup of biochemical biomarker. To evaluate fasting insulin and free fatty acids among patients with acute ischemic stroke, the blood samples employed in this investigation were randomly selected from the biomarker substudy of the registry. Patients with diabetes history or a history of antidiabetic agents or antidiabetic treatment during hospitalization or without available baseline fasting insulin or free fatty acids content on admission were excluded from this study.

### Baseline data collection

Within 24 h after admission, professional neurologists from participating institutions conducted face-to-face interviews to gather baseline information on demographics and cardiovascular risk factors. Cardiovascular risk factors included triglyceride, total cholesterol, high-density lipoprotein cholesterol, low-density lipoprotein cholesterol, body mass index, smoking status (current or ever smoker), drinking status (regular drinking, alcohol intake > 20 g/day), and a history of prior stroke, coronary heart disease, atrial fibrillation, hypertension, dyslipidemia. Body mass index was calculated as weight (kg) divided by the square of height (m^2^). At admission, qualified research coordinators directly interviewed patients to collect prestroke modified Rankin Scale (mRS) [23, 24] and National Institutes of Health Stroke Scale (NIHSS) score. According to the Trial of Org 10,172 in Acute Stroke Treatment (TOAST) criteria, stroke patients were categorized into large-artery atherosclerosis (LAA), cardioembolic stroke (CE), small-vessel occlusion (SVO), stroke of other determined cause (SOC), and stroke of undetermined cause (SUC). Demographics, medical history, medication history (antihypertensive agents, lipid-regulating agents, anticoagulants and antiplatelet agents), physical exam, primary diagnosis, laboratory testing, and risk factor evaluation were also acquired from medical records. Additionally, complications from pulmonary or urinary infections as well as medications during hospitalization were documented.

### Blood sample collection and laboratory tests

Serum samples were collected within 24 h of admission and after an overnight fast of at least 8 h to assess the fasting insulin and free fatty acids. The serum specimens were collected in serum-separation tubes and EDTA anticoagulation blood collection tube, then shipped on ice by overnight courier from each site to Beijing Tiantan Hospital, where all serum specimens were stored at − 80 °C until testing was performed. Free fatty acid levels (FFA) were measured with an enzymatic method using the OLYMPUS AU2700 automatic biochemical analyzer. Insulin levels (INS) were measured by competitive radioimmunoassay. Fasting insulin, free fatty acid, triglyceride, total cholesterol, high-density lipoprotein cholesterol, and low-density lipoprotein cholesterol were all centrally measured.

To quantify the degree of insulin resistance in adipose tissue, Adipo-IR was calculated as follows: [17, 25]

$$Adipo - IR\, = \,INS(\mu U/mL) * FFA\,\left( {mmol/L} \right)$$

INS indicates serum fasting insulin; FFA indicates serum fasting free fatty acids.

The Adipo-IR quantifies the extent of adipose tissue specific insulin resistance when considering both serum insulin and free fatty acids. The patients were then categorized into 5 groups by quintiles of Adipo-IR (Q1–Q5) for further comparisons.

### Follow-up and outcomes

Patients were followed up at 12 months after stroke onset by trained research coordinators via telephone interview. Stroke recurrence was the primary outcome in our study, which was defined as a subsequent stroke (ischemic or haemorrhagic) during 1-year follow-up period. Combined vascular events were defined as a composite of myocardial infarction, recurrent stroke (either ischemic or hemorrhagic), and vascular death, whichever occurred first. Death is defined as all-cause mortality, and each case fatality was confirmed on a death certificate from the attended hospital or the local citizen registry. Poor outcome was defined as the mRS score of 3–6. All reported events were verified by a central adjudication committee that was blinded to the study group assignments. Follow-up time was calculated from the day of enrollment in the registry project.

### Statistical analysis

Continuous variables are expressed as means and standard deviations (SD) or medians or interquartile ranges (IQR), and categorical variables are expressed as frequencies and percentages. Online Supplementary Table S1 shows baseline characteristics of individuals included and excluded. We compared the baseline characteristics of the study population using Chi-square test for categorical variables or One-Way ANOVA for continuous variables.

A proportional hazard Cox model was used to determine the relationships between the categories of Adipo-IR and the outcomes of stroke recurrence, combined vascular events, and mortality during 12-month follow-up. Adjusted hazard ratios (HR) with their 95% confidence intervals (CI) were estimated for each category of Adipo-IR, using the lowest quintile as the reference. The proportional hazard assumption was checked by tests based on Schoenfeld residuals, and the results indicated that the assumptions had not been violated. For the poor outcome at 12 months, adjusted odds ratios with their 95% confidence intervals were estimated by using a logistic regression model. Two models were used in the multivariable regression analysis after considering the covariate collinearity diagnostics (variance inflation factor for Adipo-IR and all adjustment variables is less than 5). In the first model, we adjusted for the significant variables in the univariable analysis except total cholesterol (total cholesterol was collinear with low-density lipoprotein cholesterol). In the second model, we further adjusted for prior stroke, TOAST etiology subtype, pre-stroke mRS, and NIHSS at admission to assess the association of adipose tissue specific insulin resistance with the outcomes after ischemic stroke. In addition, a sensitivity analysis was conducted to assess the influence of missing data for serum fasting insulin and fasting free fatty acids. A Cox/logistic regression model was developed using the inverse-probability-of-treatment-weighted approach to account for the missing data with adjustment for covariates in the second model [26]. A stratified analysis was conducted to examine the interaction between Adipo-IR and the prognosis of ischemic stroke patients according to TOAST subtypes. To evaluate the pattern and magnitude of associations between Adipo-IR and risk of stroke recurrence, combined vascular events, death, and poor outcome, we further performed a Cox or logistic regression model with restricted cubic splines with 5 knots (the 5th, 25th, 50th, 75th, and 95th percentiles) for the Adipo-IR (continuous variable), adjusting for covariates (model 2). The lowest value of the Adipo-IR (0.032) was treated as the reference.

All analyses were conducted with SAS 9.4 (SAS Institute Inc, Cary, NC) and R 4.1.2 (R Foundation for Statistical Computing). Two-sided *p* less than 0.05 was considered to be statistically significant.

## Results

### Study participants and characteristics

**Fig. 1:**
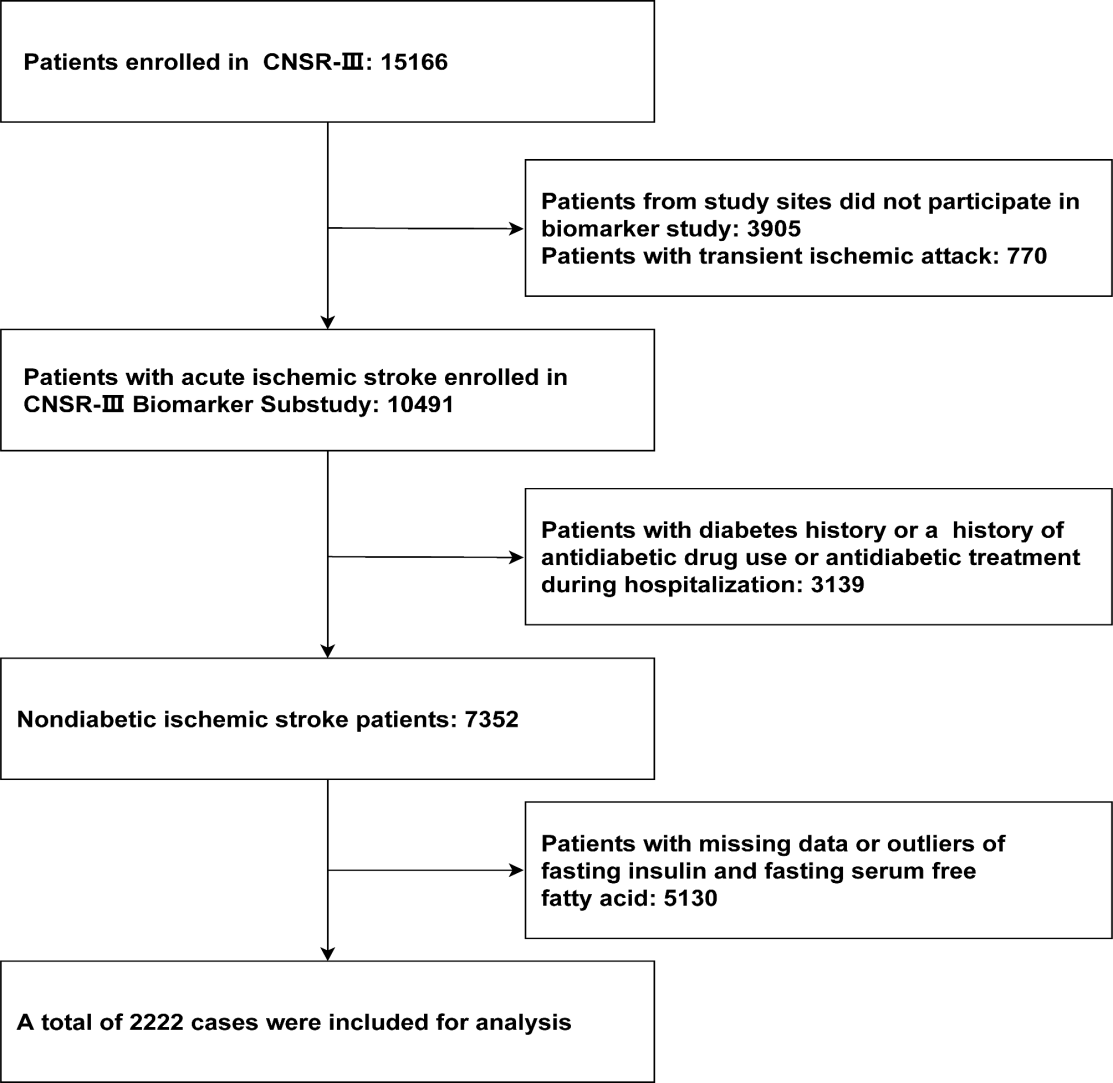
Flow chart of study population. CNSR-III, the Third China National Stroke Registry

A total of 7,352 nondiabetic patients with ischemic stroke were enrolled in the CNSR-III (Fig. 1). After excluding patients whose blood samples were not selected, 2,222 (30.2%) patients were included in this analysis. Among them, 2,169 patients had complete follow-up information. The patient included in and those excluded from this analysis were well-balanced except that those who were excluded had higher triglyceride at admission, dyslipidemia history, pulmonary infection, and lower prior stroke (Additional file 1: Table S1).

Among the 2,222 patients included, the mean age was 62.5 (SD, 11.5), and 1,533 (69.0%) patients were male. The median Adipo-IR was 5.0 (interquartile, 2.7–8.4). Table 1 shows the baseline characteristics of the included patients by quintile of Adipo-IR. Patients with higher Adipo-IR were younger, less likely to be male, and current smokers, but more likely to have a history of hypertension and dyslipidemia. Furthermore, patients with higher Adipo-IR had higher triglyceride, total cholesterol, and low-density lipoprotein cholesterol.

**Table 1:** Baseline Characteristics of the Patients by Quintile of Adipo-IR (n = 2222)

Characteristic				Adipo-IR			*P Value*
	**Total**	**Q1(N = 447)** ^a^	**Q2(N = 440)**	**Q3(N = 447)**	**Q4(N = 441)**	**Q5(N = 447)**	
**Sex (male), n (%)**	1533 (69.0)	343 (76.7)	326 (74.1)	308 (68.9)	282 (63.9)	274 (61.3)	< 0.001
**Age, y, mean (SD)**	62.5 ± 11.5	64.3 ± 10.8	63.4 ± 10.5	63.0 ± 11.5	61.2 ± 11.9	60.6 ± 12.5	< 0.001
**Body mass index, kg/m**^**2**^, **mean (SD)**	24.5 ± 3.3	23.4 ± 2.8	24.1 ± 3.1	24.4 ± 3.1	24.9 ± 3.2	25.9 ± 3.5	< 0.001
**Laboratory tests at admission**							
** TG, mmol/L, mean (SD)**	1.4 ± 0.8	1.2 ± 0.5	1.3 ± 0.6	1.4 ± 0.6	1.6 ± 0.8	1.8 ± 1.2	< 0.001
** TC, mmol/L, mean (SD)**	4.0 ± 1.0	3.8 ± 0.9	3.9 ± 0.9	4.0 ± 1.0	4.1 ± 1.1	4.3 ± 1.1	< 0.001
** HDL-C, mmol/L, mean (SD)**	1.0 ± 0.3	1.1 ± 0.3	1.0 ± 0.2	1.0 ± 0.3	1.0 ± 0.3	1.0 ± 0.3	< 0.001
** LDL-C, mmol/L, mean (SD)**	2.4 ± 0.9	2.2 ± 0.8	2.3 ± 0.8	2.4 ± 0.9	2.4 ± 0.9	2.6 ± 1.1	< 0.001
**Medical history, n (%)**							
** Prior stroke**	501 (22.5)	97 (21.7)	93 (21.1)	117 (26.2)	98 (22.2)	96 (21.5)	0.36
** Coronary heart disease**	193 (8.7)	33 (7.4)	34 (7.7)	44 (9.8)	37 (8.4)	45 (10.1)	0.50
** Atrial fibrillation**	171 (7.7)	45 (10.1)	34 (7.7)	35 (7.8)	27 (6.1)	30 (6.7)	0.22
** Hypertension**	1306 (58.8)	243 (54.4)	236 (53.6)	261 (58.4)	264 (59.9)	302 (67.6)	< 0.001
** Dyslipidemia**	106 (4.8)	11 (2.5)	11 (2.5)	29 (6.5)	18 (4.1)	37 (8.3)	< 0.001
**Current smoking, n (%)**	727 (32.7)	179 (40.0)	155 (35.2)	144 (32.2)	133 (30.2)	116 (26.0)	< 0.001
**Regular drinking, n (%)**	318 (14.3)	71 (15.9)	75 (17.0)	61 (13.6)	58 (13.2)	53 (11.9)	0.17
**Pre-stroke mRS 3–5, n (%)**	94 (4.2)	18 (4.0)	14 (3.2)	17 (3.8)	17 (3.9)	28 (6.3)	0.19
**Medication history**							
** Antihypertensive agents**	934 (42.0)	161 (36.0)	174 (39.5)	186 (41.6)	201 (45.6)	212 (47.4)	0.004
** Lipid-regulating agents**	227 (10.2)	36 (8.1)	36 (8.2)	55 (12.3)	46 (10.4)	54 (12.1)	0.09
** Anticoagulants**	17 (0.8)	3 (0.7)	2 (0.5)	7 (1.6)	1 (0.20	4 (0.9)	0.19
** Antiplatelet agents**	364 (16.4)	57 (12.8)	75 (17.0)	80 (17.9)	69 (15.6)	83 (18.6)	0.14
**NIHSS at admission, median (IQR)**	3.0 (2.0–6.0)	3.0 (2.0–6.0)	4.0 (2.0–6.0)	3.0 (2.0–6.0)	3.0 (1.0–6.0)	4.0 (2.0–6.0)	0.28
**Stroke etiology, n (%)**							0.01
** Large-artery atherosclerosis**	567 (25.5)	99 (22.1)	120 (27.3)	118 (26.4)	126 (28.6)	104 (23.3)	
** Cardioembolism**	148 (6.7)	43 (9.6)	34 (7.7)	29 (6.5)	19 (4.3)	23 (5.1)	
** Small-artery occlusion**	514 (23.1)	115 (25.7)	85 (19.3)	91 (20.4)	109 (24.7)	114 (25.5)	
** Others**	993 (44.7)	190 (42.5)	201 (45.7)	209 (46.8)	187 (42.4)	206 (46.1)	
**Urinary infection, n (%)**	22 (1.0)	5 (1.1)	7 (1.6)	7 (1.6)	2 (0.5)	1 (0.2)	0.13
**Pulmonary infection, n (%)**	102 (4.6)	18 (4.0)	17 (3.9)	21 (4.7)	27 (6.1)	19 (4.3)	0.50
**Medicines during hospitalization, n (%)**							
** Antiplatelet agents**	2149 (96.7)	435 (97.3)	420 (95.5)	434 (97.1)	430 (97.5)	430 (96.2)	0.41
** Antihypertensive agents**	958 (43.1)	156 (34.9)	155 (35.2)	191 (42.7)	233 (52.8)	223 (49.9)	< 0.001
** Lipid-lowering agents**	2138 (96.2)	428 (95.7)	423 (96.1)	427 (95.5)	430 (97.5)	430 (96.2)	0.23
** Warfarin**	54 (2.4)	9 (2.0)	13 (3.0)	10 (2.2)	9 (2.0)	13 (2.9)	0.81
** Intravenous alteplase**	231 (10.4)	30 (6.7)	53 (12.0)	53 (11.9)	56 (12.7)	39 (8.7)	0.01
**Medicine use during 1-year follow-up**							
** Antihypertensive agents**	1068 (48.1)	191 (42.7)	185 (42.0)	198 (44.3)	255 (57.8)	239 (53.5)	< 0.001
** Lipid-regulating agents**	1607 (72.3)	338 (75.6)	321 (73.0)	309 (69.1)	325 (73.7)	314 (70.2)	0.19
** Anticoagulants**	67 (3.0)	13 (2.9)	15 (3.4)	12 (2.7)	12 (2.7)	15 (3.4)	0.51
** Antiplatelet agents**	1761 (79.3)	370 (82.8)	344 (78.2)	352 (78.7)	352 (79.8)	352 (79.8)	0.14

Abbreviations: Adipo-IR, adipose tissue specific insulin resistance index; TG, triglyceride; TC, total cholesterol; HDL-C, high-density lipoprotein cholesterol; LDL-C, low-density lipoprotein cholesterol; IQR, interquartile range; mRS, modified Rankin Scale; NIHSS, National Institutes of Health Stroke Scale score

^a^ Quintiles of Adipo-IR, Q1 = < 2.28, Q2 = 2.28-4.00, Q3 = 4.01–6.05, Q4 = 6.06–9.49, Q5 = ≥ 9.50

### Association of adipose tissue specific insulin resistance with outcomes

At 12 months, 185 (8.3%) patients had recurrent stroke, 193 (8.7%) had combined vascular events, 58 (2.6%) died, and 250 (11.5%) had poor outcome. Compared with non-recurrent stroke individuals, patients with a new stroke at 12 months were more likely to be dysfunctional, have a history of prior stroke, coronary heart disease, obtain a urinary and pulmonary infection, and have a lower level of triglyceride (Additional file 1: Table S2).

Table 2 shows the 12-month prognosis after ischemic stroke across quintiles of Adipo-IR. All of the proportional hazard assumptions were met (*P* = 0.44 for stroke recurrence, *P* = 0.38 for combined vascular events, and *P* = 0.31 for death). Patients in the higher Adipo-IR quintiles were marginally associated with a higher risk of 12-month stroke recurrence and poor outcomes (*P* for trend = 0.055 and 0.09, respectively). In analysis adjusted for age, sex, body mass index, medical history, laboratory tests at admission, medication history, smoking status, and medicine use during hospitalization and follow-up (model 1), high Adipo-IR was associated with the higher risk of 12-month stroke recurrence, combined vascular events and poor outcome. Further adjustment for prior stroke, TOAST etiology subtype, pre-stroke mRS, and NIHSS at admission (model 2), we found that compared with the first quintile of Adipo-IR, the other four quintiles of the higher Adipo-IR were associated with an increased risk of 12-month stroke recurrence ( HR, 1.77; 95% CI, 1.04–3.03; *P* = 0.04; HR, 2.19; 95% CI, 1.30–3.68; *P* = 0.003; HR, 1.84; 95% CI, 1.06–3.21; *P* = 0.03; HR, 2.11; 95% CI, 1.20–3.71; *P* = 0.01, respectively) and marginally associated with an increased risk of combined vascular events ( HR, 1.60; 95%CI, 0.97–2.64; *P* = 0.07; HR, 1.91; 95% CI, 1.17–3.13; *P* = 0.01; HR, 1.62; 95% CI, 0.96–2.75; *P* = 0.07; HR, 1.80; 95% CI, 1.05–3.09; *P* = 0.03, respectively). There was no clear evidence of an association between the Adipo-IR groups and death at the 12 months in both models. Analysis that utilized the inverse-probability-of-treatment-weighted approach to account for missing data (model 3) yielded results that were consistent with the main analysis. In addition, there was no interaction between Adipo-IR and TOAST subtypes (*P* values for interaction were all > 0.05) in stratified analysis (Additional file 1: Table S3).

**Table 2:** Adjusted hazard ratios/odds ratio of outcomes at 12-month according to Adipo-IR

				Model 1^a^		Model 2 ^b^		Model 3 ^c^	
**Outcomes**	**Adipo-IR groups**	**N**	**Events, n (%)**	**HR/OR (95%CI)** ^d^	***P Value***	**HR/OR (95%CI)** ^d^	***P*** **Value**	**HR/OR (95%CI)** ^d^	***P Value***
**Stroke recurrence**	Q1 (< 2.28)	447	24 (5.4)	Ref.		Ref.		Ref.	
	Q2 (2.28-4.00)	440	39 (8.9)	1.84 (1.08–3.13)	0.03	1.77 (1.04–3.03)	0.04	1.68 (1.24–2.28)	< 0.001
	Q3 (4.01–6.05)	447	48 (10.7)	2.32 (1.38–3.90)	0.002	2.19 (1.30–3.68)	0.003	2.08 (1.55–2.80)	< 0.001
	Q4 (6.06–9.49)	441	37 (8.4)	1.93 (1.11–3.36)	0.02	1.84 (1.06–3.21)	0.03	1.82 (1.33–2.50)	< 0.001
	Q5 (≥ 9.50)	447	37 (8.3)	2.22 (1.27–3.91)	0.01	2.11 (1.20–3.71)	0.01	2.12 (1.55–2.91)	< 0.001
	Per SD			1.19 (1.02–1.39)	0.03	1.19 (1.01–1.39)	0.03	1.22 (1.12–1.33)	< 0.001
	*P* for trend				0.04		0.055		< 0.001
**Combined vascular events** ^e^	Q1 (< 2.28)	447	28 (6.3)	Ref.		Ref.		Ref.	
	Q2 (2.28-4.00)	440	41 (9.3)	1.65 (0.99–2.72)	0.052	1.60 (0.97–2.64)	0.07	1.51 (1.13–2.01)	0.01
	Q3 (4.01–6.05)	447	49 (11.0)	2.02 (1.24–3.30)	0.01	1.91 (1.17–3.13)	0.01	1.82 (1.38–2.40)	< 0.001
	Q4 (6.06–9.49)	441	38 (8.6)	1.69 (0.99–2.86)	0.051	1.62 (0.96–2.75)	0.07	1.61 (1.20–2.17)	0.002
	Q5 (≥ 9.50)	447	37 (8.3)	1.91 (1.11–3.26)	0.02	1.80 (1.05–3.09)	0.03	1.81 (1.34–2.45)	< 0.001
	Per SD			1.17 (1.02–1.37)	0.045	1.17 (0.98–1.37)	0.055	1.20 (1.10–1.31)	< 0.001
	*P* for trend				0.08		0.12		0.003
**Death**	Q1 (< 2.28)	447	12 (2.7)	Ref.		Ref.		Ref.	
	Q2 (2.28-4.00)	440	9 (2.0)	0.92 (0.37–2.29)	0.86	0.91 (0.36–2.32)	0.84	0.91 (0.54–1.54)	0.72
	Q3 (4.01–6.05)	447	16 (3.6)	1.55 (0.68–3.52)	0.30	1.72 (0.75–3.99)	0.20	1.68 (1.04–2.71)	0.03
	Q4 (6.06–9.49)	441	9 (2.0)	1.07 (0.41–2.84)	0.89	1.16 (0.43–3.14)	0.76	1.23 (0.71–2.14)	0.47
	Q5 (≥ 9.50)	447	12 (2.7)	1.77 (0.72–4.36)	0.21	1.55 (0.62–3.91)	0.35	1.53 (0.91–2.57)	0.11
	Per SD			1.07 (0.78–1.46)	0.67	1.01 (0.74–1.38)	0.95	0.99 (0.84–1.18)	0.95
	*P* for trend				0.19		0.33		0.08
**Poor outcome** ^f^	Q1 (< 2.28)	439	43 (9.8)	Ref.		Ref.		Ref.	
	Q2 (2.28-4.00)	427	47 (11.0)	1.20 (0.76–1.91)	0.44	1.19 (0.73–1.95)	0.49	1.19 (0.90–1.58)	0.21
	Q3 (4.01–6.05)	436	52 (11.9)	1.45 (0.92–2.28)	0.11	1.35 (0.83–2.19)	0.22	1.30 (0.99–1.71)	0.06
	Q4 (6.06–9.49)	435	53 (12.2)	1.72 (1.07–2.75)	0.02	1.62 (0.98–2.69)	0.06	1.62 (1.22–2.15)	0.001
	Q5 (≥ 9.50)	432	55 (12.7)	2.00 (1.23–3.24)	< 0.001	1.53 (0.91–2.56)	0.11	1.47 (1.10–1.96)	0.01
	Per SD			1.25 (1.08–1.45)	< 0.001	1.15 (0.98–1.36)	0.09	1.14 (1.04–1.25)	0.004
	*P* for trend				0.003		0.09		<0.001

Abbreviations: Adipo-IR, adipose tissue specific insulin resistance index; CI, confidence interval; HR, hazard ratio; mRS, modified Rankin Scale; NIHSS, National Institutes of Health Stroke Scale score; OR, odds ratio; TOAST, Trial of Org 10,172 in Acute Stroke Treatment

^a^ Model 1: adjusted for sex and age, body mass index, hypertension, dyslipidemia, current smoking, history of antihypertensive medications, triglyceride, high-density lipoprotein cholesterol, low-density lipoprotein cholesterol at admission, antihypertensive agents and intravenous alteplase use during hospitalization and antihypertensive agents during 1-year follow-up

^b^ Model 2: adjusted for model 1 + prior stroke, TOAST etiology subtype, pre-stroke mRS, NIHSS at admission

^c^ Model 3: model 2 using the inverse-probability-of-treatment-weighted approach to account for missing data for serum fasting insulin and fasting free fatty acids

^d^ HR for stroke recurrence, combined vascular events and death, while OR for poor outcome

^e^ Combined vascular events were defined as a composite of myocardial infarction, recurrent stroke (either ischemic or hemorrhagic) and vascular death, whichever occurred first

^f^ Poor outcome: modified Rankin Scale 3–6

**Fig. 2:**
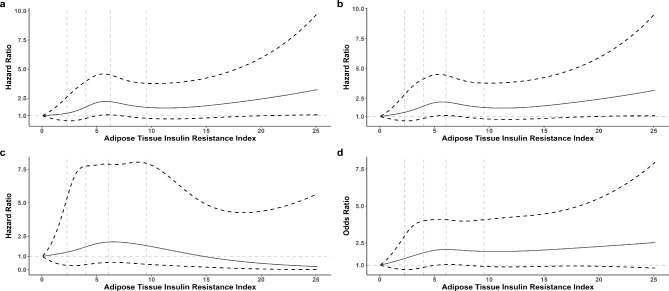
Risk of **(a)** stroke recurrence, **(b)** combined vascular events, **(c)** death, and **(d)** poor outcome according to the index of adipose tissue specific insulin resistance (Adipo-IR). The solid line indicates adjusted hazard ratio/odds ratio and the dashed lines the 95% confidence interval bands. Reference is the minimum value of Adipo-IR (0.032). The vertical dashed lines indicate the first, second, third and fourth quintile of Adipo-IR. Data were fitted using a Cox/logistic regression model of restricted cubic spline with 5 knots (the 5th, 25th, 50th, 75th, and 95th percentiles) for Adipo-IR, adjusting for potential covariates

Using a Cox/logistic regression model with restricted cubic spline, we found that a higher level of Adipo-IR was associated with an increased risk of stroke recurrence, combined vascular events, and poor outcome (Fig. 2).

## Discussion

In nondiabetic patients with ischemic stroke, adipose tissue specific insulin resistance measured by Adipo-IR was associated with 12-month risk of poor clinical outcomes, including stroke recurrence and combined vascular events in the large nationwide clinical registry. This suggests that adipose tissue specific insulin resistance may play an important role in pathologic mechanism of stroke prognosis.

Insulin resistance, a complicated metabolic reaction, was commonly estimated using absolute fasting glucose values and insulin values, without taking into account the adipose tissue specificity of insulin resistance [27]. This type of nonspecific insulin resistance has been shown to be associated with poor stroke outcome [28]. In ACROSS-China Study (Abnormal Glucose Regulation in Patients With Acute Stroke Across China) in China, insulin resistance measured by the top quartile of HOMA-IR (a homeostasis model assessment of insulin resistance) displayed an association with an increased risk of death, stroke recurrence, and poor outcome in nondiabetic patients with acute ischemic stroke [29]. The association between HOMA-IR and poor neurological outcomes was also observed in the Fukuoka Stroke Registry in Japan [12]. Although the simple and minimally invasive index of HOMA-IR has been applied in several studies for the measurement of insulin resistance, this simple surrogate marker was limited to particular study populations or measurement time, not suitable for patients with severely impaired or absent β-cell function, and did not take into account the important role in insulin resistance played by specific tissue [9]. Apart from insulin resistance in the liver and skeletal muscle, insulin resistance is also present in adipose tissue and contributes to the pathophysiology of metabolic diseases, which has not been widely appreciated [30]. In contrast, insulin resistance in adipose tissue represents the simultaneous consideration of glucose metabolism and lipid metabolism pathways, which may be an early metabolic defect in the development of whole-body insulin resistance [17].This process results in the production of an excess of free fatty acids and a number of inflammatory adipokines, which encourage ectopic fat accumulation and lipotoxicity in muscle, liver, and pancreatic cells [31], preceding insulin resistance in the liver and skeletal muscle [25, 32]. Previous research have shown the Adipo-IR may indicate adipose tissue dysfunction, and this index has shown a correlation between adipose tissue specific insulin resistance and vascular risk factors in non-stroke patients [18, 33, 34]. Our prospective analysis further added the evidence that adipose tissue specific insulin resistance was associated with an increased risk of poor outcomes in patients with acute ischemic stroke. This study indicates that the Adipo-IR may be an important indicator in clinical practice for the timely assessment of the degree of insulin resistance in stroke patients without glucose abnormalities.

Although the mechanisms of how adipose tissue specific insulin resistance affects the prognosis of stroke are unclear, there are several possible explanations. First, inflammation and fibrosis are widely present in the adipose tissue of insulin-resistant patients, and this process is to blame for more ischemic brain injuries, such as stroke and stroke recurrence [35]. Second, patients with high Adipo-IR frequently have other obesity-related risk factors such as high body mass index, triglycerides, total cholesterol, low-density lipoprotein cholesterol, and a history of hyperlipidemia, which are key players in favoring combined cardiovascular and cerebrovascular events [28, 36–38]. Third, glucose uptake and lipogenesis are diminished in adipose tissue specific insulin resistance, and lipolysis is repressed to a lesser amount. As a result, the concentration of circulating free fatty acids increases [39], causing pancreatic β-cell dysfunction via lipotoxicity [40], which has shown to be linked to stroke recurrence in non-diabetic stroke patients [41].

Several limitations must be acknowledged when interpreting the results. First, our assessment of adipose tissue specific insulin resistance did not use the gold-standard multistep pancreatic clamp technique (coupled with radiolabeled tracers) or adipose microdialysis, which are both intrusive and time-consuming [42]. Second, we recognize that residual confounding may have affected the relationship between insulin resistance and prognosis. There were no comprehensive data on physical activity, exercise, diet, or cardiorespiratory fitness in this investigation, which are considered to be lifestyles associated with glycolipid regulation [25]. Third, missing data and outliers of fasting insulin or fasting free fatty acids accounted for a larger proportion of nondiabetic stroke patients. However, the baseline characteristics between the patients included in and those excluded from the analyses were well balanced and similar findings were observed in sensitivity analysis with inverse-probability-of-treatment-weighted model to account for missing data. Fourth, follow-up by telephone was a limitation of this study and mild clinical events may be overestimated. Finally, our investigation exclusively included Chinese population, which may limit the generalization of our findings. Future study with a larger sample size among other ethnic groups is required to validate the results.

## Conclusions

In this nationwide large-scale registry, higher Adipo-IR, denoting a greater degree of adipose tissue specific insulin resistance, was associated with 12-month stroke recurrence and combined vascular events in nondiabetic patients with ischemic stroke. Our findings provide evidence on the detrimental effect of adipose tissue specific insulin resistance on stroke prognosis.

### Electronic supplementary material

Below is the link to the electronic supplementary material.

Supplementary Material 1

## Data Availability

The datasets generated during the current study are available from the corresponding author upon reasonable request.

## Abbreviations

Adipo-IR: Adipose tissue specific insulin resistance index
CE: Cardiologist
CI: Confidence interval
FFA: Free fatty acid
HDL-C: High-density lipoprotein cholesterol
HOMA-IR: Homeostasis model assessment of insulin resistance
HR: Hazard ratio
INS: Insulin
IQR: Interquartile range
LAA: Large-artery atherosclerosis
LDL-C: Low-density lipoprotein cholesterol
mRS: Modified Rankin Scale
NIHSS: National Institutes of Health Stroke Scale score
OR: Odds ratio
SOC: Stroke of other determined cause
SUC: Stroke of undetermined cause
SVO: Small-vessel occlusion
TC: Total cholesterol
TG: Triglyceride
TIA: Transient ischemic attack
TOAST: Trial of Org 10,172 in Acute Stroke Treatment criteria
