# Supplementary material for: Adipose tissue specific insulin resistance and prognosis of nondiabetic patients with ischemic stroke
Source: Diabetol Metab Syndr. 2023 Dec 2;15:246. doi: 10.1186/s13098-023-01235-2 (PMC10693091; doi:10.1186/s13098-023-01235-2)
Supplement: Supplementary file 1 — Supplementary Material 1 [file 13098_2023_1235_MOESM1_ESM.docx]

**Supplementary Materials**

**Additional file 1**

**Table S1** Baseline characteristics of individuals included and excluded

**Table S2** Baseline characteristics of patients according to 12-month recurrent stroke or not

**Table S3** Adjusted hazard ratios/odds ratios and 95%CIs for association between different Adipo-IR levels and outcomes at 12-month, separated by the stroke etiology

**Table S1** Baseline characteristics of individuals included and excluded

| **Characteristic** | **Included (N=2222)** | **Excluded  (N=5130)** | ***P* Value** |
| --- | --- | --- | --- |
| **Sex (male), n (%)** | 1533 (69.0) | 3651 (71.2) | 0.06 |
| **Age, y, mean (SD)** | 62.5±11.5 | 62.0±11.8 | 0.10 |
| **Body mass index, kg/m^2^, mean (SD)** | 24.5±3.3 | 24.5±3.3 | 0.28 |
| **Laboratory tests at admission** |  |  |  |
| **TG, mmol/L, mean (SD)** | 1.4±0.8 | 1.5±0.9 | <0.001 |
| **TC, mmol/L, mean (SD)** | 4.0±1.0 | 4.1±1.3 | 0.64 |
| **HDL-C, mmol/L, mean (SD)** | 1.0±0.3 | 1.0±0.3 | <0.001 |
| **LDL-C, mmol/L, mean (SD)** | 2.4±0.9 | 2.5±1.1 | 0.19 |
| **Medical history, n (%)** |  |  |  |
| **Prior stroke** | 501 (22.5) | 1032 (20.1) | 0.02 |
| **Coronary heart disease** | 193 (8.7) | 493 (9.6) | 0.21 |
| **Atrial fibrillation** | 171 (7.7) | 434 (8.5) | 0.27 |
| **Hypertension** | 1306 (58.8) | 3076 (60.0) | 0.34 |
| **Dyslipidemia** | 106 (4.8) | 363 (7.1) | <0.001 |
| **Current smoking, n (%)** | 727 (32.7) | 1743 (34.0) | 0.29 |
| **Regular drinking, n (%)** | 318 (14.3) | 766 (14.9) | 0.49 |
| **Pre-stroke mRS 3–5, n (%)** | 94 (4.2) | 239 (4.7) | 0.42 |
| **NIHSS at admission, median (IQR)** | 3.0 (2.0–6.0) | 3.0 (2.0–6.0) | 0.13 |
| **Stroke etiology, n (%)** |  |  | 0.08 |
| **Large-artery atherosclerosis** | 567 (25.5) | 1224 (23.9) |  |
| **Cardioembolism** | 148 (6.7) | 381 (7.4) |  |
| **Small-artery occlusion** | 514 (23.1) | 1107 (21.6) |  |
| **Others** | 993 (44.7) | 2418 (47.1) |  |
| **Urinary infection, n (%)** | 22 (1.0) | 73 (1.4) | 0.13 |
| **Pulmonary infection, n (%)** | 102 (4.6) | 303 (5.9) | 0.02 |
| **Medicine use during hospitalization, n (%)** |  |  |  |
| **Antiplatelet agents** | 2149 (96.7) | 4908 (95.7) | 0.11 |
| **Antihypertensive agents** | 958 (43.1) | 2261 (44.1) | 0.43 |
| **Lipid-lowering agents** | 2138 (96.2) | 4928 (96.1) | 0.61 |
| **Warfarin** | 54 (2.4) | 112 (2.2) | 0.51 |
| **Intravenous alteplase** | 231 (10.4) | 539 (10.5) | 0.89 |

Abbreviations: TG, triglyceride; TC, total cholesterol; HDL-C, high-density lipoprotein cholesterol; LDL-C, low-density lipoprotein cholesterol; IQR, interquartile range; mRS, modified Rankin Scale; NIHSS, National Institutes of Health Stroke Scale score; TOAST, Trial of Org 10172 in Acute Stroke Treatment.

**Table S2** Baseline characteristics of patients according to 12-month recurrent stroke or not

| **Characteristic** | **Non-recurrent stroke**  **(N=2037)** | | **Recurrent stroke**  **(N=185)** | | ***P* Value** |
| --- | --- | --- | --- | --- | --- |
| **Sex (male), n (%)** | 1410 (69.2) | 123 (66.5) | | 0.44 | |
| **Age, y, mean (SD)** | 62.5±11.5 | 63.2±11.8 | | 0.40 | |
| **Body mass index, kg/m^2^, mean (SD)** | 24.6±3.3 | 24.1±3.1 | | 0.07 | |
| **Laboratory tests at admission** |  |  | |  | |
| **TG, mmol/L, mean (SD)** | 1.4±0.8 | 1.3±0.6 | | 0.01 | |
| **TC, mmol/L, mean (SD)** | 4.1±1.0 | 3.9±1.0 | | 0.11 | |
| **HDL-C, mmol/L, mean (SD)** | 1.0±0.3 | 1.0±0.3 | | 0.64 | |
| **LDL-C, mmol/L, mean (SD)** | 2.4±0.9 | 2.3±0.9 | | 0.37 | |
| **Medical history, n (%)** |  |  | |  | |
| **Prior stroke** | 443 (21.7) | 58 (31.4) | | 0.003 | |
| **Coronary heart disease** | 165 (8.1) | 28 (15.1) | | 0.001 | |
| **Atrial fibrillation** | 151 (7.4) | 20 (10.8) | | 0.10 | |
| **Hypertension** | 1200 (58.9) | 106 (57.3) | | 0.67 | |
| **Dyslipidemia** | 99 (4.9) | 7 (3.8) | | 0.51 | |
| **Current smoking, n (%)** | 668 (32.8) | 59 (31.9) | | 0.80 | |
| **Regular drinking, n (%)** | 291 (14.3) | 27 (14.6) | | 0.91 | |
| **Pre-stroke mRS 3–5, n (%)** | 81 (4.0) | 13 (7.0) | | 0.048 | |
| **NIHSS at admission, median (IQR)** | 3.0 (2.0–6.0) | 4.0 (2.0–6.0) | | 0.12 | |
| **Stroke etiology, n (%)** |  |  | | 0.02 | |
| **Large-artery atherosclerosis** | 507 (24.9) | 60 (32.4) | |  | |
| **Cardioembolism** | 135 (6.6) | 13 (7.0) | |  | |
| **Small-artery occlusion** | 487 (23.9) | 27 (14.6) | |  | |
| **Others** | 908 (44.6) | 85 (45.9) | |  | |
| **Urinary infection, n (%)** | 17 (0.8) | 5 (2.7) | | 0.01 | |
| **Pulmonary infection, n (%)** | 87 (4.3) | 15 (8.1) | | 0.01 | |
| **Medicine use during hospitalization, n (%)** |  |  | |  | |
| **Antiplatelet agents** | 1975 (97.0) | 174 (94.1) | | 0.10 | |
| **Antihypertensive agents** | 883 (43.3) | 75 (40.5) | | 0.65 | |
| **Lipid-lowering agents** | 1965 (96.5) | 173 (93.5) | | 0.13 | |
| **Warfarin** | 47 (2.3) | 7 (3.8) | | 0.21 | |
| **Intravenous alteplase** | 208 (10.2) | 23 (12.4) | | 0.34 | |

Abbreviations: TG, triglyceride; TC, total cholesterol; HDL-C, high-density lipoprotein cholesterol; LDL-C, low-density lipoprotein cholesterol; IQR, interquartile range; mRS, modified Rankin Scale; NIHSS, National Institutes of Health Stroke Scale score; TOAST, Trial of Org 10172 in Acute Stroke Treatment.

**Table S3** Adjusted hazard ratios/odds ratios and 95%Cis for association between different Adipo-IR levels and outcomes at 12-month, separated by the stroke etiology

|  |  | **Large-artery atherosclerosis** | | | | **Cardioembolism** | | | | **Small-artery occlusion** | | | | **Others** | | | |  |
| --- | --- | --- | --- | --- | --- | --- | --- | --- | --- | --- | --- | --- | --- | --- | --- | --- | --- | --- |
| **Outcomes** |  | **N** | **n (%)** | **HR/OR (95%CI) ^a,b.^** | **P value** | **N** | **n (%)** | **HR/OR**  **(95%CI) ^a,b.^** | **P value** | **N** | **n (%)** | **HR/OR (95%CI) ^a,b.^** | **P value** | **N** | **n (%)** | **HR/OR (95%CI) ^a,b.^** | **P value** | **P for interaion** |
| **Stroke recurrence** | Q1 (<2.28) | 99 | 9 (9.1) | Ref. |  | 43 | 2 (4.7) | Ref. |  | 115 | 7 (6.1) | Ref. |  |  |  | Ref. |  |  |
|  | Q2 (2.28-4.00) | 120 | 14 (11.7) | 1.27 (0.53-3.07) | 0.59 | 34 | 3 (8.8) | 2.05 (0.21-20.34) | 0.54 | 85 | 2 (2.4) | 0.46 (0.09-2.34) | 0.35 | 190 | 6 (3.2) | 3.09 (1.22-7.84) | 0.02 | 0.72 |
|  | Q3 (4.01-6.05) | 118 | 15 (12.7) | 1.46 (0.60-3.57) | 0.40 | 29 | 4 (13.8) | 14.07 (1.12-176.84) | 0.04 | 91 | 7 (7.7) | 1.63 (0.52-5.17) | 0.40 | 201 | 20 (10.0) | 3.10 (1.23-7.83) | 0.02 |  |
|  | Q4 (6.06-9.49) | 126 | 11 (8.7) | 1.06 (0.41-2.74) | 0.91 | 19 | 3 (15.8) | - | - | 109 | 5 (4.6) | 0.87 (0.21-3.57) | 0.85 | 209 | 22 (10.5) | 3.50 (1.34-9.13) | 0.01 |  |
|  | Q5 (≥9.50) | 104 | 11 (10.6) | 1.08 (0.39-2.99) | 0.88 | 23 | 1 (4.3) | 1.01 (0.02-44.65) | >0.99 | 114 | 6 (5.3) | 1.06 (0.27-4.14) | 0.94 | 187 | 18 (9.6) | 4.17 (1.61-10.81) | 0.003 |  |
| **Combined vascular events ^c^** | Q1 (<2.28) | 99 | 10 (10.1) | Ref. |  | 43 | 4 (9.3) | Ref. |  | 115 | 7 (6.1) | Ref. |  | 190 | 7 (3.7) | Ref. |  |  |
|  | Q2 (2.28-4.00) | 120 | 14 (11.7) | 1.14 (0.49-2.66) | 0.77 | 34 | 3 (8.8) | 1.03 (0.16-6.66) | 0.97 | 85 | 2 (2.4) | 0.46 (0.09-2.34) | 0.35 | 201 | 22 (10.9) | 3.03 (1.27-7.21) | 0.01 | 0.74 |
|  | Q3 (4.01-6.05) | 118 | 15 (12.7) | 1.32 (0.56-3.12) | 0.53 | 29 | 4 (13.8) | 5.62 (0.78-40.53) | 0.09 | 91 | 7 (7.7) | 1.63 (0.52-5.17) | 0.40 | 209 | 23 (11.0) | 2.81 (1.18-6.69) | 0.02 |  |
|  | Q4 (6.06-9.49) | 126 | 11 (8.7) | 0.94 (0.37-2.36) | 0.89 | 19 | 3 (15.8) | 8.34 (0.71-97.43) | 0.09 | 109 | 5 (4.6) | 0.87 (0.21-3.57) | 0.85 | 187 | 19 (10.2) | 3.27 (1.33-8.05) | 0.01 |  |
|  | Q5 (≥9.50) | 104 | 11 (10.6) | 0.95 (0.36-2.55) | 0.92 | 23 | 1 (4.3) | 1.59 (0.07-34.68) | 0.77 | 114 | 6 (5.3) | 1.06 (0.27-4.14) | 0.94 | 206 | 19 (9.2) | 3.57 (1.45-8.79) | 0.01 |  |
| **Death** | Q1 (<2.28) | 99 | 4 (4.0) | Ref. |  | 43 | 2 (1.7) | Ref. |  | 115 | 2 (4.7) | Ref. |  | 190 | 4 (2.1) | Ref. |  |  |
|  | Q2 (2.28-4.00) | 120 | 1 (0.8) | 0.27 (0.03-2.69) | 0.26 | 34 | 0 (0.0) | - | - | 85 | 0 (0.0) | - | - | 201 | 8 (4.0) | 1.77 (0.40-7.71) | 0.45 | 0.71 |
|  | Q3 (4.01-6.05) | 118 | 5 (4.2) | 1.64 (0.36-7.39) | 0.52 | 29 | 2 (2.2) | - | - | 91 | 3 (10.3) | - | - | 209 | 6 (2.9) | 1.28 (0.28-5.89) | 0.75 |  |
|  | Q4 (6.06-9.49) | 126 | 4 (3.2) | 1.18 (0.21-6.67) | 0.85 | 19 | 0 (0.0) | - | - | 109 | 3 (15.8) | - | - | 187 | 2 (1.1) | 0.79 (0.12-5.01) | 0.80 |  |
|  | Q5 (≥9.50) | 104 | 3 (2.9) | 0.65 (0.10-4.34) | 0.66 | 23 | 1 (0.9) | - | - | 114 | 1 (4.3) | - | - | 206 | 7 (3.4) | 1.88 (0.44-8.00) | 0.40 |  |
| **Poor outcome ^d^** | Q1 (<2.28) | 99 | 11 (11.1) | Ref. |  | 43 | 3 (7.0) | Ref. |  | 115 | 10 (8.7) | Ref. |  | 190 | 19 (10.0) | Ref. |  |  |
|  | Q2 (2.28-4.00) | 120 | 16 (13.3) | 1.62 (0.64-4.12) | 0.31 | 34 | 3 (8.8) | 1.21 (0.17-8.60) | 0.85 | 85 | 2 (2.4) | 0.11 (0.01-0.97) | 0.05 | 201 | 26 (12.9) | 1.41 (0.67-2.94) | 0.37 | 0.47 |
|  | Q3 (4.01-6.05) | 118 | 19 (16.1) | 1.87 (0.75-4.68) | 0.18 | 29 | 3 (10.3) | 1.25 (0.14-11.17) | 0.84 | 91 | 4 (4.4) | 0.51 (0.13-1.98) | 0.33 | 209 | 26 (12.4) | 1.35 (0.65-2.79) | 0.42 |  |
|  | Q4 (6.06-9.49) | 126 | 20 (15.9) | 2.13 (0.83-5.45) | 0.11 | 19 | 5 (26.3) | 5.31 (0.60-46.93) | 0.13 | 109 | 6 (5.5) | 0.64 (0.16-2.58) | 0.53 | 187 | 22 (11.8) | 1.69 (0.78-3.67) | 0.18 |  |
|  | Q5 (≥9.50) | 104 | 23 (22.1) | 2.6 (0.99-6.75) | 0.051 | 23 | 5 (21.7) | 6.30 (0.66-60.20) | 0.11 | 114 | 3 (2.6) | 0.25 (0.04-1.51) | 0.13 | 206 | 24 (11.7) | 1.33 (0.61-2.91) | 0.47 |  |

Abbreviations: Adipo-IR, adipose tissue specific insulin resistance index; CI, confidence interval; HR, hazard ratio; mRS, modified Rankin Scale; NIHSS, National Institutes of Health Stroke Scale score; OR, odds ratio; TOAST, Trial of Org 10172 in Acute Stroke Treatment.

**^a^** Adjusted for sex and age, body mass index, hypertension, dyslipidemia, current smoking, history of antihypertensive medications, triglyceride, high-density lipoprotein cholesterol, low-density lipoprotein cholesterol at admission, antihypertensive agents and intravenous alteplase use during hospitalization and antihypertensive agents during 1-year follow-up, prior stroke, TOAST etiology subtype, pre-stroke mRS, NIHSS at admission.

^b^ HR for stroke recurrence, combined vascular events and death, while OR for poor outcome.

^c^ Combined vascular events were defined as a composite of myocardial infarction, recurrent stroke (either ischemic or hemorrhagic) and vascular death, whichever occurred first.

^d^ Poor outcome: modified Rankin Scale 3-6.
